# Cervical small cell neuroendocrine tumor mutation profiles via whole exome sequencing

**DOI:** 10.18632/oncotarget.14098

**Published:** 2016-12-21

**Authors:** Soo Young Cho, Minhye Choi, Hyo-Jeong Ban, Chang Hyeon Lee, Soojun Park, HanKyeom Kim, Young-Sik Kim, Young Seek Lee, Ji-Yun Lee

**Affiliations:** ^1^ Laboratory of Developmental Biology and Genomics, College of Veterinary Medicine, Research Institute for Veterinary Science BK21, Program for Veterinary Science, Seoul National University, Seoul 08826, Republic of Korea; ^2^ Department of Pathology, College of Medicine, Korea University, Seoul 02841, Republic of Korea; ^3^ Division of Molecular and Life Sciences, Hanyang University, Ansan 15588, Republic of Korea; ^4^ Division of Bio-Medical Informatics, Center for Genome Science, National Research Institute of Health, Centers for Disease Control and Prevention, Choongchung-Buk-do 28159, Republic of Korea; ^5^ Life Science Solutions Group, Thermo Fisher Scientific Corporation, Seoul 06349, Republic of Korea; ^6^ Bio-Medical IT Research Department, ETRI, Yusoeng-gu, Daejeon 34129, Republic of Korea

**Keywords:** cervical small cell neuroendocrine tumor, ATRX, ERBB4, AKT/mTOR, whole exome sequencing

## Abstract

Cervical small cell neuroendocrine tumors (CSCNETs) are rare, aggressive neuroendocrine tumors (NETs). Reliable diagnostic and prognostic CSCNET markers are lacking, making diagnosis and prognosis prediction difficult, and treatment strategies limited. Here we provide mutation profiles for five tumor-normal paired CSCNETs using whole exome sequencing (WES). We expanded our assessment of frequently mutated genes to include publicly available data from 55 small intestine neuroendocrine tumors, 10 pancreatic neuroendocrine tumors, 42 small cell lung cancers, six NET cell lines, and 188 cervical cancers, along with our five CSCNETs. We identified 1,968 somatic mutations, including 1,710 missense, 106 nonsense, 144 splice site, 4 lncRNA, 3 nonstop, and 1 start codon mutation. We assigned functions to the 114 most frequently mutated genes based on gene ontology. *ATRX*, *ERBB4*, and genes in the Akt/mTOR pathway were most frequently mutated. Positive cytoplasmic ERBB4 immunohistochemical staining was detected in all CSCNET tumors tested, but not in adjacent normal tissues. To our knowledge, this study is the first to utilize WES in matched CSCNET and normal tissues to identify somatic mutations. Further studies will improve our understanding of how *ATRX* and *ERBB4* mutations and AKT/mTOR signaling promote CSCNET tumorigenesis, and may be leveraged in novel anti-cancer treatment strategies.

## INTRODUCTION

Neuroendocrine tumors (NETs) are a heterogeneous group of neoplasms derived from the diffuse neuroendocrine system. Cervical small cell NET (CSCNET) is an extremely rare neuroendocrine carcinoma of the cervix, accounting for up to 2% of all cervical carcinomas [1–3]. Based on the Surveillance Epidemiology and End Results (SEER) database, the mean annual CSCNET incidence in the United States from 1977 to 2003 was 0.06 cases per 100,000 women [4]. CSCNETs are morphologically and clinically categorized as poorly differentiated, high grade NETs, while typical and atypical carcinoids are categorized as well differentiated, low/intermediate grade cervical NETs. CSCNETs are clinically aggressive, with unfavorable outcomes and limited treatment strategies. As a result of early metastases to lymph nodes and distant organs, along with vascular invasion, CSCNET is usually not confined to the cervix at the time of diagnosis [5–7]. Disease diagnosis and prognosis prediction are limited by a lack of reliable standardized diagnostic or prognostic markers.

In this study, we performed whole exome sequencing (WES) using five CSCNETs and paired normal tissues to identify key mutations and disrupted pathways. Even with a limited sample number due to disease rarity, we identified frequent mutations in *ATRX* and *ERBB4*, and in the Akt/mTOR signaling pathway. Our findings suggest the possibility of a new anti-cancer treatment strategy using ERBB4-Akt/mTOR inhibitors in CSCNET patients.

## RESULTS

### CSCNET mutation profiles

**Figure 1:**
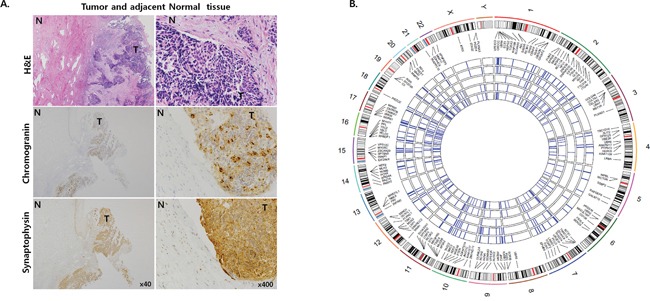
Representative histopathological analyses and somatic mutation signatures from five CSCNETs Representative (case 10) H&E and IHC staining for chromogranin and synaptophysin in tumor and adjacent normal tissues **A.** Tumor tissue stains positive for chromogranin and synaptophysin. Magnifications: x40 (left) and x400 (right). T: tumor tissue; N: adjacent normal tissue. Circos plots showing 114 recurrently mutated genes within 463 missense mutations found in ≥3 CSCNETs **B.** Five inner circles represent five CSCNETs each and blue lines represent nonsynonymous mutations in each sample. Outer circle represents chromosome with cytoband. Colors in the cytoband indicate: centromere (red), high gene density (black), low gene density (white), and gene empty loci (sky blue).

Immunohistochemical (IHC) staining with chromogranin A and synaptophysin confirmed neuroendocrine differentiation of 16 CSCNETs (Figure 1A, Table 1). Of these 16 samples, the somatic mutation signatures from five tumor-adjacent normal paired CSCNETs were profiled via WES with 136.83X mean sequencing coverage across the targeted bases (Supplementary Table 1), and minimum read depths of 14 in tumors and 8 in normal tissues. We identified 1,968 somatic mutations, including 1,710 missense, 106 nonsense, 144 splice site, 4 lncRNA, 3 nonstop, and 1 start codon mutation, and compared our results with Cosmic data (http://cancer.sanger.ac.uk/cosmic/gene/; accessed 09202016) (Supplementary Table 2). Asian-specific variations were removed using normal lymphocyte DNA datasets from the 1000 Genome Project ASN. Germline and common polymorphisms were filtered via comparison to matching adjacent normal tissue samples and sequences in the Single Nucleotide Polymorphism database (dbSNP). CSCNET mutation spectrums were characterized by a predominance of C>T/G>A transitions, as observed in other solid tumors (Supplementary Figure 1) [8]. A total of 463 mutation sites were identified from 114 recurrently mutated genes (≥50%) in more than three samples (Figure 1B, Supplementary Table 3).

**Table 1:** Clinicopathological characteristics of 16 CSCNETs

Case	Reviewed Diagnosis	Age(year)	Tumor size(cm)	FIGOstage	Treatment	Metastasis	LVI	LNmetastasis	Follow up(months)	Clinicaloutcome	IHC			
											Ki-67(%)	CgA	Syn	ERBB4
1	smcc	51	1.4×2.0	IB1	OP	brain	No	No	34	Died	5	+	+	+++
2*	smcc	45	2.5×1.0	IB1	OP	No	Yes	No	9	Alive	57.9	+	**+**	+++
3	smcc	39	2.5×2.5	IV	No OP	Vertebra	Yes	No	9	Died	90	-	+	+
4	smcc	40	3.5×3.2	III	No OP	No	Yes	Yes	101	Alive	63.5	-	+	+
5	smcc	51	2×1.8	IB1	OP, CTx	No	Yes	Yes	166	Alive	1	-	+	+
6	smcc,AIS withintestinal metaplasia	51	1.2×0.6	IB1	OP, CTx	No	Yes	No	160	Alive	47.5	+	+	+
7	smcc	28	1.0×0.36	IB1	OP, CTx	No	No	Yes	152	Alive	0	+	+	++
8	smcc	37	2.3×1.5	IB1	OP, CTx	Lung	Yes	Yes	11	Died	1.6	+	+	++
9	smcc	47	5.3×2.7	IB2	OP, CTx	No	Yes	Yes	59	Alive	72.5	+	+	+++
10*	smcc	46	1.2×0.8	IB1	OP, CTx,RTx	No	Yes	No	12	Alive	50.1	+	+	+++
11*	smcc	35	1.5×1.5	IB1	OP, CTx	No	Yes	No	30	Died	79.6	+	+	+++
12	smcc+rosette	45	2.0×2.2	IB1	OP	Lung, pancreas, bone	Yes	No	60	Alive	41.3	+	+	++
13*	smcc+rosette	48	4.5×1.6	IIA	OP	No	Yes	Yes	100	Alive	35	-	+	++
14	smcc	47	1.5×1.5	IB1	OP	no	Yes	No	60	Alive	67.8	+	+	++
15	smcc+rosette	47	1×1.5	IV	No OP,CTx, RTx	Bone, liver	Yes	Yes	29	Died	60	+	+	++
16*	smcc	33	2.1×1.0	IV	No OP,CTx, RTx	Lung	Yes	No	17	Alive	1	+	+	++

Abbreviations: *Normal and tumor tissues analyzed via WES; CNET: small cell neuroendocrine tumor; FIGO: Fédération Internationale de Gynécologieetd’ Obstétrique; LVI: lymphovascular invasion; LN: lymph node; IHC: immunohistochemistry; CgA: chromogranin A; Syn: synaptophysin; OP: radical hysterectomy; CTx: chemotherapy; RTx: radiotherapy.

Gene set analysis (GSA) was performed using the Database for Annotation, Visualization, and Integrated Discovery (DAVID). We assigned functions to the 114 frequently mutated genes based on gene ontology [9]. Genes with functions in small GTPase mediated signal transduction, forebrain development, and protein kinase B/AKT signaling were enriched (P<0.001; Supplementary Table 4). Signal transduction pathways facilitated by small GTPases, like those in the RAS/Rho family, play important roles in multiple cellular and developmental processes, including differentiation, cell division, cytoskeletal dynamics, and cell survival. Deregulation of small GTPase-mediated transduction by various mechanisms is associated with multiple cancers [10]. Association with pathways involved in forebrain development may explain the origin of CSCNETs, which derive from a neuroendocrine cell that performs neuroendocrine integration. Protein kinase B signaling, also known as AKT signaling, promotes cell survival and growth in response to extracellular signals. This pathway, along with mTOR signaling, has been linked to a range of cancers, including cervical cancer and NETs [11–16].

In our study, genes with recurrent mutations were detected in five CSCNETs, and other NETs were analyzed by combining mutation data from 118 samples representing 10 pancreatic neuroendocrine tumors (panNETs) [17], 42 small cell lung cancers (SCLCs) [18], 55 small intestine neuroendocrine tumors (SINETs) [19], six NET cell lines [20] and our five CSCNETs (Supplementary Table 5A–5E). We selected a recurrent mutation rate of ≥5% in other NETs and ≥50% (in ≥3 samples) in our CSCNETs. Two genes (*PLEC*, *OBSCN*) were mutated in CSCNET, SCLC and NET cell lines, one gene (*TSC2*) was mutated in CSCNET, panNET and NET cell lines, five genes (*PLEC*, *VPS13A*, *OBSCN*, *KIAA1109* and *PCNX*) were mutated in CSCNET and SCLC, and six genes (*KANK1*, *TSC2*, *NF1*, *DYNC1H1*, *PTEN* and *TTC21B*) were mutated in CSCNET and panNET. However, no single gene was mutated in every NET (Supplementary Figure 2).

### Recurrent *ATRX* mutation in CSCNETs

Mutation profiles have been described in panNET, SINET, and SCLC using genome wide mutation analysis [17–21]. Mutations in *ATRX*, previously identified in SINET [19] and panNET with incidences of 17.65–30.23% [17, 21], were found in four CSCNET samples in this study (Supplementary Table 6). These mutations include p.R250X (case 12), p.N281S (case 16), p.G1042R (case 2) and p.R2387G (case 10), which were not identified in other published NETs [17, 19, 21] or Cosmic data (http://cancer.sanger.ac.uk/cosmic/gene/; accessed 09202016) (Supplementary Table 2). We predicted the functional impact of each annotated mutation site *in silico* to estimate domain activity (mutationassessor.org) (Supplementary Figure 3, Supplementary Table 6 and Supplementary Table 7) [22]. Two annotated mutation sites (p.R250X and p.N281S) were located in the ATRX-DNMT3-DNMT3L (ADD) domain. p.R250X produced a stop codon and p.N281S had a low predicted functional impact. The two other annotated mutation sites, p.G1042R and p.R2387G, had neutral predicted functional impacts. However, R2387G is in a highly conserved region.

### Recurrent *ERBB4* mutation and expression in CSCNETs

**Figure 2:**
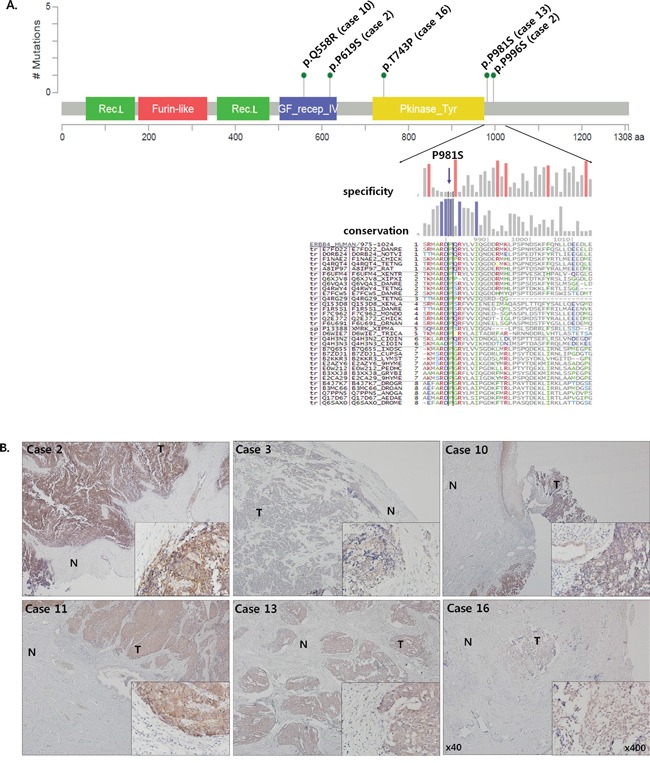
EBRR4 mutation and protein expression Functional domains containing five ERBB4 mutation sites from four CSCNETs **A.** Predictions for each mutation site indicated that p.P981S is highly conserved across species (mutationassessor.org). Representative IHC staining for ERBB4 **B.** Tumors, but not adjacent normal tissues, were positive for cytoplasmic ERBB4 expression. Cases 2, 10, and 11 exhibited the strongest expression, cases 13 and 16 showed medium expression, and case 3 showed the lowest expression. Original magnifications are x40 (big image) and x400 (small image). T: tumor tissue; N: adjacent normal tissue

ERBB4 is a member of the epidermal growth factor receptor (EGFR) subfamily. When bound by ligands like neuregulin, ERBB4 activates numerous downstream pathways, including Ras/MAPK/ERK and PI3K/AKT signaling (via mTOR), to regulate both normal cellular processes and cancer development and progression [23–25]. We identified five ERBB4 mutations, three of which (p.P6619S, p.P981S and p.P996S) were annotated in Cosmic (http://cancer.sanger.ac.uk/cosmic/gene/; accessed 09202016) (Supplementary Table 2) and in four samples. We predicted functional impact for each annotated mutation site *in silico* (mutationassessor.org) (Figure 2A, Supplementary Table 6 and Supplementary Table 7) [22]. Three annotated mutation sites were located in functional domains, but were predicted to have low or neutral impacts. One of these mutations (p.T743P: case 16) was located in the protein binding site kinase domain, and two (p.Q558R: case 10, and p.P619S: case 2) were located in the growth factor (GF)-receptor IV domain site. Two additional mutations were located in the intracellular domain (p.P981S: case 13, and p.P996S: case 2) and were predicted to have moderate functional impacts. ERBB4 protein expression was examined by IHC staining in all 16 CSCNETs, including the 11 samples in which WES was not performed. All 16 tumor samples exhibited greater positive cytoplasmic ERBB4 staining as compared to adjacent normal tissues (Figure 2B, Table 1). Five samples, including cases 2 and 10, with *ERBB4* mutations, and case 11, without *ERBB4* mutation, showed the strongest expression. Seven cases, including cases 13 and 16, with *ERBB4* mutations, exhibited modest expression. The remaining four cases showed minimal expression. We could not correlate *ERBB4* mutation status with protein level, because mutation analysis in 11 samples could not be performed due to sample quality and/or quantity. However, higher ERBB4 expression correlated with lower OS by Kaplan-Meier analysis, although this correlation was not statistically significant, likely due to small sample size (Supplementary Figure 4).

### Recurrent Akt/mTOR signaling pathway gene mutation in CSCNETs

**Figure 3:**
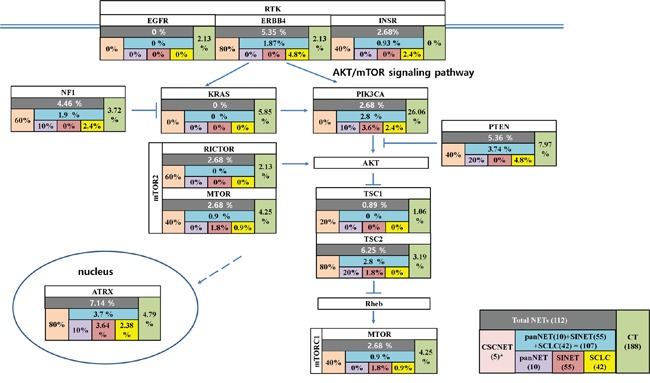
Recurrently mutated RTK-Akt/mTOR pathway genes found in CSNCETs, other NETs, and CT (): number of samples. *Indicated gene mutation rate was high, likely due to low sample number.

The Akt/mTOR signaling pathway is a recurrent driver pathway promoting NETs [13, 14, 16]. GSA indicated enrichment of genes in the mTOR (GO:0032006) and Akt (GO:0051896) signaling pathway in CSCNETs in our study (P<0.01). The dynamic mutation profiles of Akt/mTOR signaling pathway genes downstream of the receptor tyrosine kinase (RTK) were assessed across several NETs, including panNET, SINET, SCLC, and CSCNETs. *ERBB4*, *NF1*, *PTEN*, *RICTOR*, and *TSC1/2* were recurrently mutated in our CSCNETs (Figure 3, Supplementary Table 6).

### Cervical tumor (CT) signatures in CSCNET mutations

**Figure 4:**
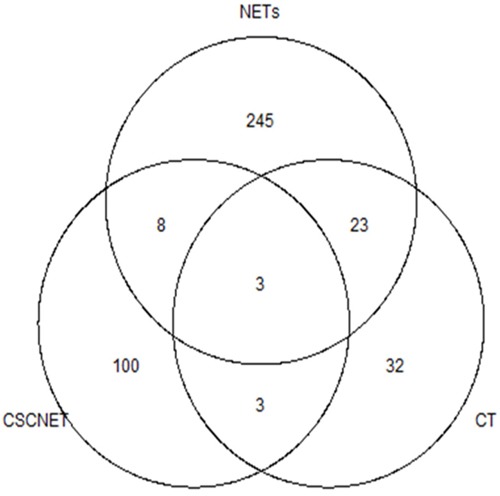
Venn diagram of frequently mutated gene(s) shared across CSCNETs, NETs and CT Recurrently mutated genes were selected at ≥5% in CTs and other NETs, and ≥50% (≥3 samples) in our CSCNETs. Mutated genes are listed in Supplementary Table 8A.

CT genomic studies have identified recurrent somatic driver mutations in *PIK3CA*, *KRAS*, *TP53*, *ERBB2* and *MAPK1* [26, 27] that were not found in our CSCNETs. However, the recurrent somatic *TSC2*, *NF1* and *PTEN* mutations identified in our CSCNETs were also identified in NETs, but not in CTs (Figure 4, Supplementary Table 8). Although mutations in some genes, including in *FLNC*, *MUC5B*, *PCLO*, *PLEC*, *OBSCN* and *VPS13A*, were found in our CSCNETs and in CTs or NETs, they are not likely to drive CT or NET development or progression, because their recurrence rates are very low compared to those of predicted driver genes. This confirms that the CSCNET origin and genetic background are likely to be different from those of CT, and more similar to those of NETs.

## DISCUSSION

This study provides the first report of the CSCNET mutation landscape as determined by WES. A limitation of our study is the small number (five) of CSCNET cases, due to extremely low disease incidence. We identified recurrent mutations in *ATRX*, *EBRR4*, and in AKT/mTOR signaling pathway genes, such as *NF1*, *PTEN*, *RICTOR* and *TSC2* in our CSCNETs. We found that the CSCNET mutation profile was more similar to that of other NETs as compared to CTs, indicating that genomic mutation may be correlated with tumor cell origin.

The *ATRX* mutation was highlighted in panNET, and identified in SINET, SCLC, and CT. In our CSCNETs, four out of five samples analyzed contained an *ATRX* mutation, although more samples must be tested to affirm this incidence rate. ATRX transcriptionally regulates genes activated during interphase and chromosomal segregation in mitosis [28]. Clinical implications of ATRX mutations are contradictory, and mutations were associated with better prognosis in Caucasian patients, but poorer prognosis in Chinese patients via panNET [17, 21]. These findings suggest that *ATRX* mutations may be frequent in NETs and underscore the need for understanding the common pathogenesis of NETs. The effects of ATRX mutations in CSCNET must still be explored.

*ERBB4* mutations (found in 4/5 CSCNETs analyzed) and ERBB4 protein levels (positive in 11/11 CSCNETs) were examined. Due to small sample sizes, we could not precisely correlate *ERBB4* mutation site and predicted functional impact with ERBB4 protein levels. However, IHC staining showed that cytoplasmic ERBB4 expression only occurred in tumor tissues and not in adjacent normal tissues. Somatic *ERBB4* mutations have been reported in various cancers [29–31], and studies attempting to assess the impacts of these mutations have suggested both oncogenic and tumor suppressor functions for ERBB4 [22, 23, 30–33]. One model suggests that signaling downstream of wild type ERBB4 is enriched in the JAK/STAT pathway, whereas mutant ERBB4 signals more commonly via the PI3K/AKT pathway, constituting a cellular shift from a program of differentiation to that of proliferation [23, 24]. This suggests that ERBB4 gain or loss of function mutations, and other mechanisms up- or downregulating *ERBB4*, may affect downstream PI3K/AKT and mTOR signaling, which appear to be the key pathways activated in our CSCNETs. Members of the EGFR subfamily are frequently mutated oncogenes, and many are amenable therapeutic targets in various cancers, including non-small cell lung cancer and breast cancer [22]. ERBB4 may be an effective therapeutic target in CSCNETs, and further studies are necessary to better understand the clinical implications of ERBB4 expression changes.

Along with *ATRX* and *ERRB4* mutations, high mutation incidences in AKT/mTOR signaling pathway components, such as *NF1*, *PTEN*, *RICTOR*, and *TSC2*, were observed in our CSCNETs. NF1, PTEN and TSC1/2 are tumor suppressors that inhibit PI3K/AKT signaling through the TSC complex, which is a critical negative regulator of mTORC1 [12, 34]. NF1, PTEN and TCS1/2 mutations have been reported both in familial syndromes, such as type 1 neurofibromatosis, Cowden syndrome, and tuberous sclerosis complex, and in sporadic NETs [35]. RICTORs are key partnering proteins that modulate mTOR, and RICTOR mutations have been identified in SCLC [18]. AKT activation through the mTOR-RICTOR complex was reported in various tumor types [16, 36–38]. Aberrant mTOR signaling, through altered mTOR pathway component expression or activation, has been associated with prognosis in panNETs, SINET, gastric NETs, and SCLC [14, 39, 40–43]. Targeting mTOR signaling has emerged as an effective therapeutic strategy for the management of advanced NETs [15, 16, 43]. Our results suggest new options for CSCNET treatment, similar to strategies for treating other NETs, which include AKT/mTOR pathway inhibitors.

To our knowledge, this study is the first to utilize WES in matched CSCNET and normal tissues to identify somatic mutations. We identified two frequently mutated genes, *ATRX* and *ERBB4*, and implicated AKT/mTOR signaling in CSCNET tumorigenesis. Future studies will help us better understand how these mutations and signaling pathways promote CSCNET development and progression, and how they may be applied in new anti-cancer treatment strategies.

## MATERIALS AND METHODS

### Tumor sample collection

This study included 16 formalin fixed paraffin embedded (FFPE) CSCNET tissue blocks diagnosed and collected between 1997 and 2012 in South Korea. All 16 hematoxylin and eosin (H&E)-stained slides were reviewed by pathologists to confirm histological diagnoses and classifications, which were performed according to the cervical NET criteria set in the 1997 workshop of the College of American Pathologists and the National Cancer Institute [1]. Clinicopathological data and Ki-67 indexes are described in Table 1. We were unable to collect information regarding HIV infection status. Mean patient age was 43.2 years (range 27–52) and eleven cases (68.9%) showed >50% Ki-67 index. Approval for this study was obtained from the institutional Ethics Committee of Korea University (IRB No. KU-IRB-14-88-A-1) and a waiver of informed consent was granted.

### Immunohistochemical (IHC) analysis

Five of 16 tumor samples, for which paired adjacent normal tissues were available, were used to perform WES. Five FFPE paired tumor and adjacent normal tissue blocks were cut into 4μm sections, which were deparaffinized in xylene and hydrated by immersion in a graded ethanol series. Antigen retrieval was performed in a microwave by placing sections in epitope retrieval solution (0.01 M citrate buffer, pH 6.0, or 10 mM ethylenediaminetetraacetic acid, pH 8.0) for 20 min, and endogenous peroxidase was blocked via immersion in 0.3% hydrogen peroxide for 10 min. IHC staining was performed using the Dako Auto stainer plus Universal Staining System (Dako Cytomation, Carpinteria, CA, USA) with a Chem Mate DAKO En Vision detection kit (Dako Cytomation). Antibodies against chromogranin A (DAKO, Glostrup, Denmark), synaptophysin (Ventana, Roche, Tucson, USA), Ki-67 (monoclonal, MIB-1 clone, 1:50, DAKO), and ERBB4 (Santa Cruz Biotechnology, Santa Cruz, CA, USA) were used.

### DNA isolation

FFPE tissue blocks were cut into 10μm sections on slide glass. Tumor and normal tissues were visualized and separately scraped with a razor blade into 1.5 mL tubes. Scraped sections were deparaffinized twice for 5 min in xylene, rehydrated in series of 100%, 96%, and 70% ethanol for 30 sec each, stained with hematoxylin for 30 sec, rinsed with water, and incubated overnight in 1 M NaSCN at 37°C to remove crosslinks. After sample pre-treatment, DNA was isolated using the QIAamp DNA extraction kit (Qiagen, USA) according to the manufacturer's instructions. DNA samples were quantified using the Qubit^®^ dsDNA HS Assay Kit (Life Technologies, Cat. no. Q32851). A total of 100 ng isolated DNA was treated with uracil-DNA glycoslyase (UDG, New England BioLabs, Inc.) according to the manufacturer's instructions and stored at 4°C before use in the subsequent target amplification reactions.

### Ion ampliSeq^TM^ exome library preparation

An Ion AmpliSeq^TM Exome^ library was constructed using the Ion AmpliSeq™ Exome Kit (Life Technologies, Part #4487084 Rev. B.0) as per the manufacturer's protocol. Briefly, 100 ng of UDG-treated DNA was amplified for multiplex PCR with each of 12 primer-pools. The amplicons, which were partially digested primer sequences, were ligated to the Ion Torrent adapters P1 and the Ion Xpress^TM^ Barcode using DNA ligase. Adapter-ligated products were then purified using AMPure XP reagent (Beckman Coulter, Brea, CA, USA), and PCR-amplified for five cycles. The resulting library was purified using AMPure XP reagent (Beckman Coulter). 200–350 base read libraries were selected for the Pippin Prep™ instrument (Sage Science) using 2% agarose gel cassettes (Sage Science). The size-selected library was purified using AMPure XP reagent (Beckman Coulter), and library concentration and size were determined using the Agilent 2100 BioAnalyzer and the Agilent BioAnalyzer DNA High-Sensitivity LabChip (Agilent Technologies).

### Ion proton sequencing

Sample emulsion PCR, emulsion breaking, and enrichment were performed using the Ion PI^TM^ Template OT2 200 Kit v3 (Life Technologies, Part #4488318 Rev. B.0) according to the manufacturer's instructions. Equal molar ratios of multiple barcoded libraries were combined for one Ion PI^TM^ v2 chip. Two pooled Ion AmpliSeq^TM^Exome libraries were loaded onto a single Ion PI^TM^ v2 chip. Five pooled Ion AmpliSeq™ Transcriptome libraries were loaded onto a single Ion PI^TM^ v2 chip. Subsequent emulsion PCR and enrichment of the pooled library sequencing beads was performed using the Ion OneTouch^TM^ system (Life Technologies) according to the manufacturer's protocol within approximately seven hours. Finally, sequencing (520 flows) was performed with the Ion PI^TM^ v2 chip using the Ion PI^TM^ Sequencing 200 Kit v3 (Life Technologies, Part #4488315 Rev. B.0) on the Ion Proton^TM^ sequencer (Life Technologies).

### Processing of whole-exome sequencing data

After the FastQC step, trimmed FASTQ reads were aligned to human reference genome version 19 (hg19) using the TMAP mapping program with default parameters [44]. The results were sorted and compressed in BAM format using Picard SortSam. In accordance with manufacturer's instructions, duplicates were not removed from AmpliSeq data [45]. Local realignment around indels and recalibration were performed by GATK [46]. Recalibrated BAM files were used to call variants with MuTect software (version 1.1.4) using default parameters [47]. Nonsynonymous mutations within each sample were visualized by circos plot using the RCircos library (http://www.ncbi.nlm.nih.gov/pubmed/23937229). The RCircos plot produced a human chromosome ideogram heatmap with five sample tracks for mutations. We used the chromosome ideogram tables from the UCSC genome browser (https://genome.ucsc.edu/). The chromosome ideogram is shown in high-resolution to plot gene locations. Tools in the Database for Annotation, Visualization, and Integrated Discovery (DAVID) were used to assess gene ontology enrichment and to identify biological pathway categories associated with somatically mutated genes [9]. Sequence data for CSCNET samples used in this study were deposited in the European Nucleotide Archive under accession number, PRJEB12274.

### Recurrent mutations in CTs and NETs

The mutated genes in the NETs dataset are from a recent WES study of 55 SINETs [19], 10 panNETs [17], 42 SCLCs [18], six NET cell lines [20] and our five CSCNETs. The mutated CT dataset is from 188 samples from The Cancer Genome Atlas (TCGA). Publicly available somatic variant calls in mutation annotation format (MAF) files were used in this study. All MAF files were downloaded from TCGA cBioPortal or supplementary tables from each study. TCGA Pan-cancer analysis provided the distributions of mutation frequencies, types, and contexts across 12 tumor types [48]. In the TCGA study, only genes mutated in at least 5% of tumors were analyzed. With this cutoff percentage, mutated genes were selected with ≥5% recurrent rates from other NET and CT studies. We used ≥50% (≥3 samples) recurrent mutation as a cutoff for our CSCNETs. We also added significantly reported genes from each study. Selected gene mutations were nonsynonymous. Venn diagram analysis shows co-occurrence among NETs, and mutated gene overlap between CT, NETs, and CSCNETs.

## SUPPLEMENTARY MATERIALS FIGURES AND TABLES




