# Supplementary material for: Cervical small cell neuroendocrine tumor mutation profiles via whole exome sequencing
Source: Oncotarget. 2016 Dec 21;8(5):8095–104. doi: 10.18632/oncotarget.14098 (PMC5352385; doi:10.18632/oncotarget.14098)
Supplement: Supplementary file 1 [file oncotarget-08-8095-s001.pdf]

Cervical small cell neuroendocrine tumor mutation profiles via whole exome sequencing

SUPPLEMENTARY FIGURES AND TABLES

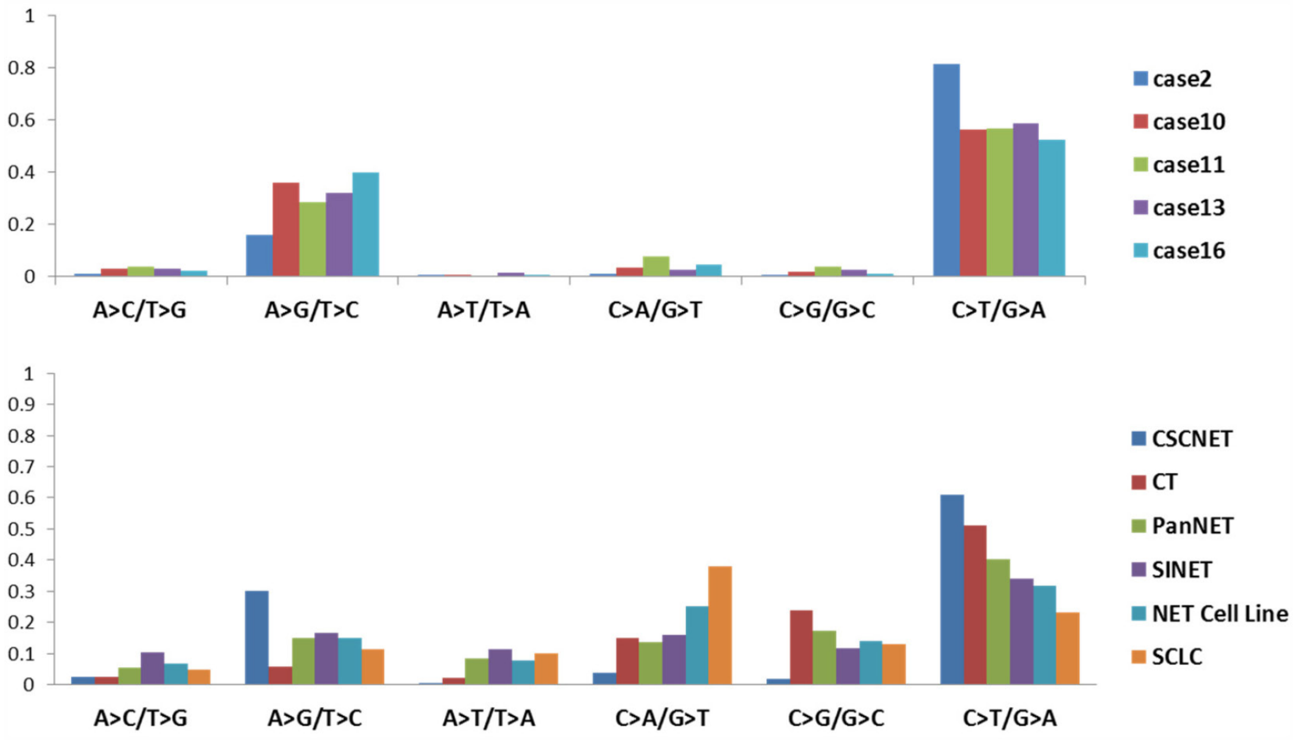

Supplementary Figure 1: Distribution of somatic substitution patterns within five CSCNETs A. Comparison of somatic mutation patterns across all NETs including panNET, SINET, SCLC, NET cell lines, our CSCNETs, and CT **B.**

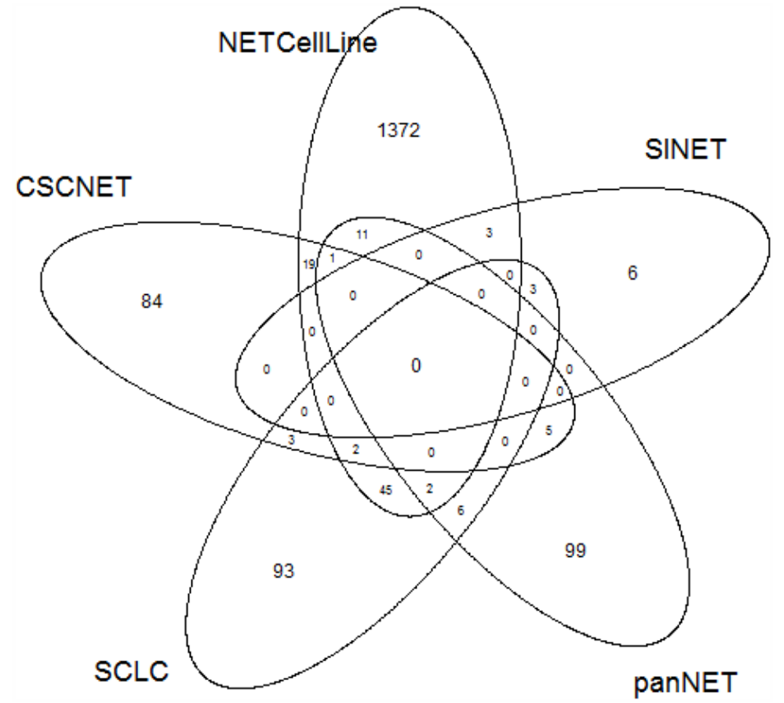

Supplementary Figure 2: Venn diagram of recurrently mutated genes shared between CSCNETs (≥50%; ≥3 samples) and other NETs (≥5%), including SCLC, panNET, SINET, and NET cell lines. No recurrently mutated gene was shared by all NETs.

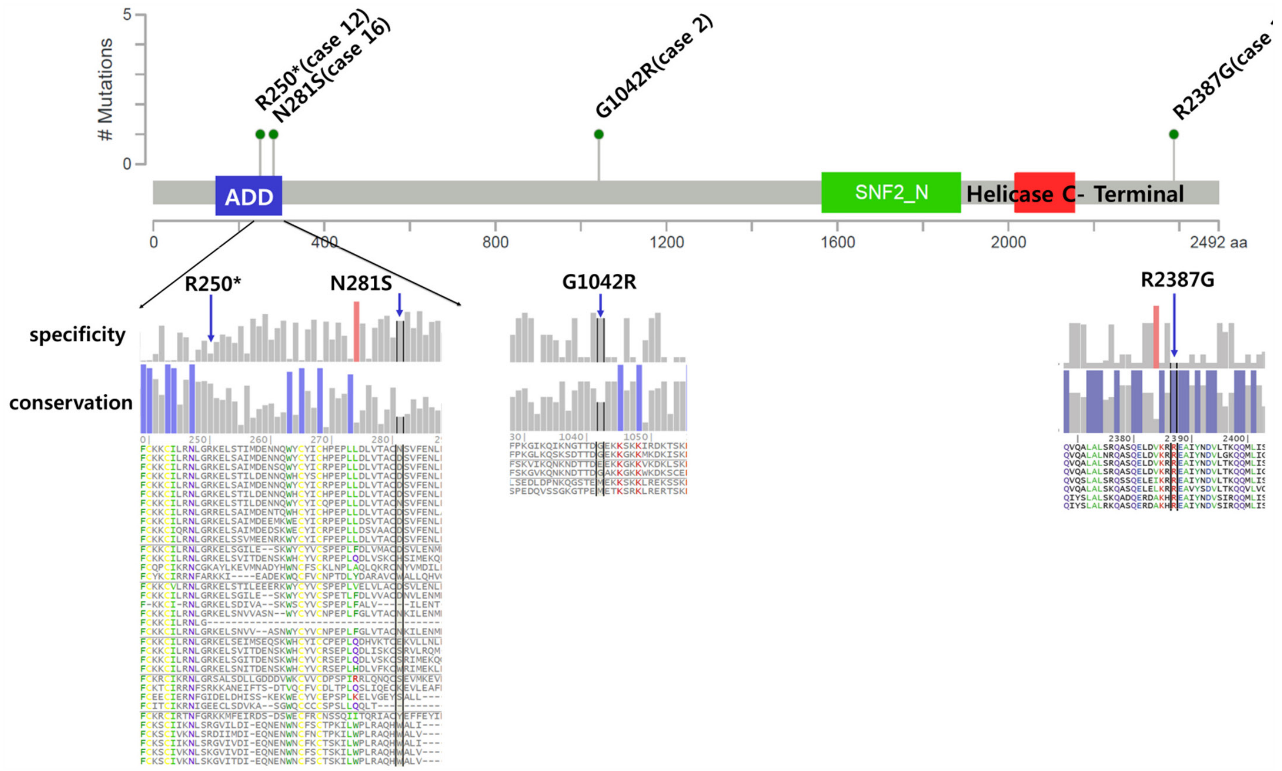

Supplementary Figure 3: Functional domain annotation of four ATRX mutation sites from four CSCNETs. Two annotated mutation sites (p.R250X and N281S) were located in the ATRX-DNMT3-DNMT3L (ADD) domain. p.R250X produced a stop codon, while p.N281S had a low predicted functional impact. p.G1042R and p.R2387G had neutral predicted functional impacts, even though p.R2387G is in a highly conserved region.

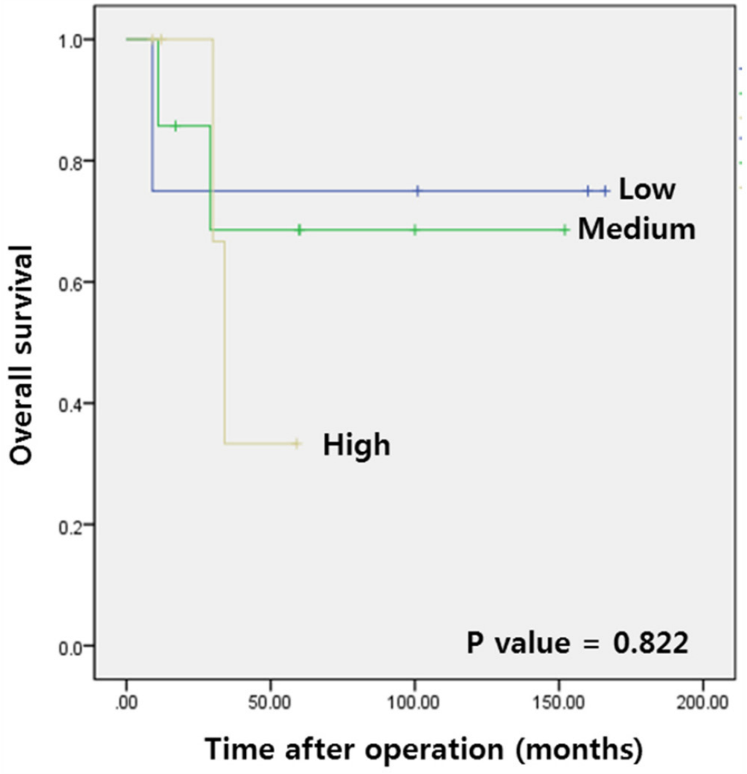

Supplementary Figure 4: Kaplan-Meier survival curve showing a consistent, but statistically insignificant ERBB4 expression correlation with overall survival.

Supplementary Table 1: Sequencing coverage and quality metrics for five CSCNETs

See Supplementary File 1

Supplementary Table 2: Complete list of somatic mutations in five CSCNETs

See Supplementary File 1

Supplementary Table 3: 463 mutation sites in 114 recurrently mutated genes (≥50% in ≥3 samples) from five CSCNETs

See Supplementary File 1

Supplementary Table 4: Gene set analysis of 114 frequently mutated genes in five CSCNETs

See Supplementary File 1

Supplementary Table 5: Recurrently mutated genes shared across NETs, including our CSCNETs A., panNET B., SCLC C., SINET D., and NET cell lines E. *Mutated genes were selected with a ≥5% recurrent rate from other NET samples and ≥50% (≥3 samples) from our CSCNETs

See Supplementary File 1

Supplementary Table 6: Selected genes mutated in five CSCNETs

See Supplementary File 1

Supplementary Table 7: Predicted functional impact of ATRX and ERBB4 amino acid substitutions

See Supplementary File 1

Supplementary Table 8: Recurrently mutated genes shared in CT, other NETs and our CSCNETs A. None were shared by all of the NETs. Percentages of recurrently mutated genes shared across CTs, other NETs and our CSCNETs B. *Mutated genes were selected with a ≥5% recurrent rate from CTs and other NETs, and ≥50% (≥3 samples) from our CSCNETs

See Supplementary File 1
